# PD-L1-centric whole blood-based immune signature profiles of tuberculosis patients during therapy

**DOI:** 10.3389/fimmu.2026.1829438

**Published:** 2026-06-12

**Authors:** Johanna Eggeling, Martina Sester, Christoph Lange, Jan Heyckendorf, Barbara Kalsdorf, Anna M. Mandalakas, Andrew R. DiNardo, David Lewinsohn, Dagmar Schaub, Tina Schmidt, Eva Tolosa, Maja Reimann, Patricia M. Sánchez Carballo

**Affiliations:** 1 Research Center Borstel, Leibniz Lung Center, Division of Clinical Infectious Diseases, Borstel, Germany; 2 German Center for Infection Research (DZIF), Tuberculosis Unit, Partner Site Hamburg-Lübeck-Borstel-Riems, Borstel, Germany; 3 Respiratory Medicine & International Health, Lübeck University, Lübeck, Germany; 4 Pediatric Oncology and Rheumatology, Schleswig-Holstein University Hospital, Kiel, Germany; 5 Neonatology, Pediatric Pneumology and Neuropediatrics, Schleswig-Holstein University Hospital, Kiel, Germany; 6 Department of Transplant and Infection Immunology, Saarland University, Homburg, Germany; 7 Global Tuberculosis Program, Division of Global Health, Baylor College of Medicine and Texas Children´s Hospital, Houston, TX, United States; 8 Schleswig-Holstein University Hospital, Internal Medicine I, Leibniz Lung Clinic, Kiel, Germany; 9 Airway Research Center North (ARCN), German Center for Lung Research (DZL), Kiel, Germany; 10 Pulmonary, Allergy and Critical Care Medicine, Oregon Health and Science University, Portland, OR, United States; 11 Institute of Immunology, Hamburg Center for Translational Immunology, University Medical Center Hamburg-Eppendorf, Hamburg, Germany; 12 German Center for Child and Adolescent Health (DZKJ), Hamburg, Germany

**Keywords:** biomarker, flow cytometry, infectious disease, machine learning, personalized medicine, programmed cell death ligand 1, risk assessment

## Abstract

This prospective study investigated whole blood-based immune cell biomarkers for pulmonary tuberculosis (TB) immunoprofiling. Blood samples from 34 healthy controls and 51 tuberculosis patients were analyzed at three timepoints: Prior to therapy (T0), after 14 days of therapy (T1), and at the end of treatment (Te). Using multiparameter flow cytometry, 386 immune cell populations were analyzed. Predictive models were developed using two machine learning algorithms. A TB5_LF change_ score, which was based on five cell populations, effectively distinguished tuberculosis patients from controls (AUC = 0.89) and tuberculosis patients before and at the end of treatment (AUC = 0.92). Similarly, the TB5_Lasso_ score distinguished tuberculosis patients from controls (AUC = 0.91) and tuberculosis patients before and at the end of treatment (AUC = 0.93) but was inversely correlated with disease severity (r=–0.43). Both scores included PD-L1^+^CD80^-^ neutrophils and PD-L1^+^HLA-DR^+^ CD4^+^ lymphocytes, highlighting PD-L1-associated and Th1-related immune signatures as candidate biomarkers for TB immunoprofiling. These findings are exploratory and hypothesis-generating, providing a basis for future studies evaluating their potential utility in diagnostic and therapy-monitoring contexts.

## Introduction

The World Health Organization (WHO) reported 10.7 million newly diagnosed and 10.8 million estimated incident tuberculosis (TB) cases in 2024, with the highest number since global TB surveillance began in 1995 (1). With 1.23 million related deaths, TB is the leading cause of death by a single microbial pathogen worldwide (1). Beyond treatment efficacy and vaccine development, the End TB Strategy, launched in 2014, emphasized the need to intensify research efforts to develop novel diagnostic tools to address the TB epidemic effectively (2). In 2023, the United Nations reiterated the importance of the End TB Strategy’s goals, highlighting the need to ensure that at least 90% of the estimated TB cases are diagnosed and treated with quality-assured methods (3).

Currently, the detection of *Mycobacterium tuberculosis* DNA via nucleic acid amplification techniques (NAATs) or bacterial culture from sputum remains the reference standard for the diagnosis of pulmonary TB, the most common clinical presentation of this disease. Usually, several weeks pass from sputum sampling to the detection of *M. tuberculosis* in liquid or solid media because of the slow growth rate of the bacteria (4). The most commonly used NAAT for the detection of *M. tuberculosis*, GeneXpert Ultra, is less than 90% sensitive in adults with *M. tuberculosis* detected in culture and much lower in children (5, 6).

Nonsputum-based diagnostics provide significant advantages for early treatment initiation and monitoring, especially at the primary healthcare level in high-incidence TB areas, as specimen collection is less invasive and faster, while the need for sophisticated laboratories with high biosafety standards is obviated. Previous research has identified aerosols, saliva, urine, stool and blood as promising sample materials for detecting TB-related components from both the host and the pathogen (7).

Blood-based diagnostics focus primarily on measuring the immune response to TB-specific antigens (e.g., interferon-γ release assays; IGRAs) but also on detecting circulating mycobacterial components (e.g., cell-free DNA) and blood biomarkers through multiomic assays (8). Artificial intelligence (AI) and machine learning techniques applied to blood cell populations show promise for improving the interpretation of complex data, potentially increasing specificity and sensitivity by identifying novel immune signatures (9, 10).

In this study, we aimed to investigate novel whole blood-based biomarkers via machine learning and mathematical modeling of longitudinal flow cytometric data from healthy adult controls and pulmonary TB patients before and after TB treatment.

## Materials and methods

### Study design and cohort

This two-step prospective observational study was conducted at the Clinical Tuberculosis Center (ClinTB) of the German Center for Infection Research (DZIF), Medical Clinic of the Research Center Borstel, Germany, from 2015-2020. Human samples and clinical data from the study participants were collected and processed in accordance with ethical review board approval No AZ 12–233 at Lübeck University, Germany.

The study cohort consisted of healthy controls and adult participants with culture-confirmed pulmonary TB. Tuberculosis was identified via GeneXpert MTB/ULTRA (Cepheid, Sunnyvale, CA, USA) performed on sputum at baseline and subsequent confirmation of *M. tuberculosis* growth in liquid and solid cultures. The study cohort was HIV-negative. The BCG vaccination and IGRA status was not assessed. From all the study participants, we built an identification cohort of healthy controls and TB patients matched by age ± five years, sex, and smoking status to reduce potential confounding during model development. Such matching was not feasible in the independent validation cohort, which was therefore used as an external, demographically heterogeneous validation dataset.

### Flow cytometry analysis of whole blood cell surface markers

Multiparameter flow cytometry analysis of cell surface marker expression in fresh peripheral venous whole blood was performed prior to antibiotic therapy initiation, following two weeks of treatment and at the end of therapy. Seven different panel assays, each with nine antibodies, were developed to investigate cell lineages and antigenic targets of interest. The panels were accordingly labeled “Subset”, “APC-like”, “Innate-like”, “T helper”, “T effector”, “T regulatory” and “T exhaustion” (**Supplementary Figures S1**–**S7**; **Supplementary Table S1**). The antibodies were diluted in a solution of sodium azide phosphate-buffered saline (Az-PBS, Merck, US, 8.22335, 1 g/L; MERCK, US, D 1408 (10x), w/o Ca; w/o Mg, pH 7.2-7.4; respectively), and a total of 50 μL of Az-PBS-diluted antibody solution was added to one polystyrene round-bottom tube (PS tube, Falcon, 352054) per panel. Heparin-anticoagulated peripheral venous blood from the study participants was obtained in 10 mL vacutainer tubes (BD, US, No 367526) and processed within a maximum of 24 h after blood collection. A total of 100 μL of whole blood was added to each of the seven antibody-containing PS tubes, which were subsequently vortexed and incubated for 30 minutes in the dark at 20 °C for cell surface staining. 2 mL of erythrocyte lysis buffer (Quicklysis, Cytognos, 14190144) was added to each tube, which was vortexed for 10 seconds and again incubated for 10 minutes in the dark at 20 °C. Incubation was followed by vortexing and centrifugation (670 g/20 °C/10 min). The supernatants were discarded. The cells were washed with 2 mL of Az-PBS, vortexed and centrifuged (670 g/20 °C/10 min). The supernatants were discarded. Next, 150 μL of Az-PBS and 150 μL of 3% (w/w)-paraformaldehyde (PFA, MERCK, US, 1.04005) were added to each tube, vortexed for 10 seconds and stored at 4 °C. Data were acquired on a LSRII flow cytometer (BD, US) via FACSDiva software (BD, US, version 8.0.0). The goal was to record 50.000 CD3^+^ events or 100.000 CD45^+^ events (where the CD3 antibody was not present), or the complete sample was measured when the goal was not achieved. Total data were recorded for the subsets, APC-like and Innate-like panels, and only CD3^+^ events were recorded in the T-cell panels. The quality maintenance of the detectors was assured by bead-based performance controls and followed-up by Fluorescence Cytometry Core Facility at Research Center Borstel, Germany. The data were analyzed with FlowJo for Mac OS X (BD, US, version 10.10.0), and R (version 4.2.2). FCS cleaning was performed via the FlowAI plugin, and the analysis was performed on the fraction of good events. Populations were reported as a percentage of the parental population and were considered not measurable when the parental population was less than 100 cells.

### Statistical analysis

R software (version 4.4.1 to 4.4.2.) was used for the statistical analyses. The experimental and clinical data were imported and exported via the openxlsx, readxl and writexl packages (Microsoft Excel for Mac OS, US, version 16.89.1). The data were processed and analyzed using the base, dlmnet, dplyr, graphics, grDevices, grid, methods, missForest, mosaic, pROC, purrr, randomForest, stats, and utils packages. For plotting and visualization, we used circlize, ComplexHeatmap, cowplot, ggplot2, ggpubr, ggrepel, ggroc, gridExtra and qgraph. Missing data were imputed via random forest-based methods and the use of 100 trees and ten iterations.

The initial comparisons between T0 and Te were calculated using the Benjamini-Hochberg-corrected Wilcoxon rank-sum test. Further comparisons between the T0, T1, Te and HC groups were performed using the Bonferroni-corrected Wilcoxon rank-sum test. The statistical significance cutoff was set at an alpha level of 0.05. Fold changes were calculated as the ratio of median values between the groups.

Flow cytometric data measured at baseline and at the end of therapy in TB patients were used as predictive features for random forest model building (**Supplementary Figure S8**). The training sets consisted of the identification cohort, while the validation sets consisted of the independent validation cohort. All preprocessing steps, including missing data imputation, feature selection, and model tuning, were strictly restricted to the identification cohort. The validation cohort was not involved in any step of model development and was used exclusively for independent performance evaluation. In the first model approach, blood cell populations were selected via LASSO regression (elastic net alpha = 1), and the minimum lambda was used. To assess the robustness of feature selection, we performed bootstrap resampling of the identification cohort (500 iterations).

The selected variables were embedded in a random forest model, and further reduction steps based on the Akaike information criterium (AIC) and variable importance were performed iteratively by the cutoff of the variable importance, resulting in 5 populations in the final model. In the second model approach, the five blood cell populations with the largest log-fold change between baseline and the end of therapy in the identification cohort were used for predictive feature selection. No additional scaling or normalization was applied prior to random forest modeling, as random forest models are invariant to monotonic transformations and do not require feature scaling. For LASSO-based feature selection, the predictors were standardized internally by glmnet using its default standardization procedure. Model calibration was assessed in the validation cohort by comparing the predicted probabilities with the observed outcomes using calibration plots and calculation of the Brier score. This was performed to evaluate the agreement between the predicted and observed event frequencies.

Both random forest models were tested via receiver operating characteristic (ROC) curves with respect to their ability to distinguish between different time points and between patients and healthy controls in the validation cohort by calculating the area under the curve (AUC). Model discrimination was interpreted on the basis of AUC values using commonly applied thresholds (<0.6 poor, 0.6–0.7 fair, 0.7–0.8 acceptable, 0.8–0.9 good, >0.9 excellent). The final models were predictively applied to all observations of the identification and validation cohorts. As a result, the calculated probabilities of being at the start or end of therapy were applied. The calculated probabilities are understood as a score.

### Development of a composite clinical disease severity score

A composite disease severity score (DSS) was developed as follows: The patients were clustered on the basis of bacterial load and clearance using the Ward D method, and two clusters were formed as a result. As surrogates, we used the baseline time to sputum culture positivity (TTP), the TTP after 14 days of therapy, the absolute change in TTP over these 14 days, and the time to sputum culture conversion (TTCC). A total of 20 clinical variables were used to create a descriptive model that could differentiate between the culture-based disease severity clusters. We assigned binary values (0 or 1) to the 20 baseline clinical parameters, with the lower-risk category consistently scored as 0. An exception was made for smear grade, which was scored from 0 to 3 on the basis of increasing bacterial burden (negative +/- = 0, + = 1, ++ = 2, +++ = 3). The smear microscopy results were graded on the basis of the number of acid-fast bacilli (AFB) observed under 1000× magnification. Smears were considered negative if no AFB were seen in 100 fields. A scanty or +/– grade was assigned when 1–9 AFB were seen per 100 fields. A grade of + indicated 10–99 AFB per 100 fields, ++ corresponded to 1–10 AFB per field in at least 50 fields, and +++ was assigned when more than 10 AFB were seen per field in at least 20 fields. The parameters included age (<65/≥65 years), sex (female/male), BMI (≥18/<18 kg/m²), prior TB history (no/yes), presence of lung cavities (no/yes), bilateral disease (no/yes), extrapulmonary TB (no/yes), alcohol abuse (no/yes), smoking (no/yes), immunosuppressive medication use (no/yes), type 1 diabetes mellitus (no/yes), baseline fever (no/yes), leukocytosis (≤/>12×10^9^/L), leukocytopenia (≥/<4×10^9^/L), thrombocytosis (≤/>450×10^9^/L), thrombocytopenia (≥/<150×10^9^/L), low hematocrit (>/≤30%), anemia (≥/<10 g/dL), and elevated creatinine (</≥2 mg/dL). Alcohol abuse was defined as self-reported regular or heavy alcohol consumption or a documented history of alcohol-related health or social issues. Smoking was defined as a cumulative lifetime smoking of >1 pack-year (py). A py was defined as smoking the equivalent of 20 cigarettes (one pack) per day for one year, corresponding to a lifetime consumption of more than 7,300 cigarettes (more than 365 packs). Immunosuppressive medication use included TNF antagonists, JAK/STAT inhibitors, and corticosteroids. Baseline fever was defined as an orally measured body temperature ≥38 °C. The maximum likelihood method was used to minimize the number of variables. This left seven variables in a model that should describe the culture-based disease severity cluster: alcohol abuse status, active smoking status, baseline smear grade, presence of lung cavities, baseline fever, leukocytosis, and anemia. These seven variables formed the basis of the descriptive clinical disease severity score. One point is awarded for each of the above binary risk factors, with the baseline smear score ranging from 0 to 3 points. This results in a clinical disease severity score (DSS) that reflects culture-related disease severity independently of culture and can assume an integer value between 0 and 9.

### Text editing

Rubriq software (formerly named Curie, American Journal Experts, AJE, US) and ChatGPT software (OpenAI, US, version 4o) were used for American English proofreading and syntax editing. After using these tools, the authors reviewed and edited the content as needed and take full responsibility for the content of the publication.

## Results

### Study populations: identification and validation cohorts

The study identification cohort (IC) included 20 adult TB patients whose blood cell populations were analyzed prior to the initiation of antituberculosis therapy at baseline (T0) and at the end of successful therapy (Te) to evaluate their potential as predictive features for treatment success. The IC was used for initial model training. An additional analytic timepoint on day 14 of therapy (T1) was included to assess early normalization of selected variables from the final scoring model and to evaluate their potential utility as treatment monitoring tools. To assess not only the immune response during the course of therapy but also the potential diagnostic performance of the models, we included 20 healthy controls (HCs) matched by age (± five years), sex, and smoking status (**Table 1**). All IC participants were HIV-negative at baseline and reported no comorbidities.

**Table 1:** Characteristics of the study identification cohort.

Cohort characteristics	Identification cohort			
	TB patients		Healthy controls	
Total number (N) Values	20		20	
	$\bar{\text{x}}$/N	ICR/%	$\bar{\text{x}}$/N	ICR/%
Age (Years)	32.0	25.5 – 43.5	33.0	27.8 – 44.0
Sex (Male)	10	50.00%	10	50.00%
M/XDR therapy	9	45.00%	NA	
Therapy duration (Days)	507	287 -612	NA	
Longer therapy as WHO recommended	15	75.00%	NA	
Smoking	9	45.00%	6	30.00%

A separate validation cohort (VC) of 31 adult TB patients and 14 adult HCs who were not matched for demographic characteristics was used to evaluate the scores (**Table 2**). The TB patients differed from the HCs in terms of age (mean=37 and mean=44.5; p=0.016) and sex distribution (74.2% and 14.3% males; p=0.00063) but not in smoking status (51.6% and 28.6% smokers; p=0.26). The proportion of individuals with drug-resistant TB was similar between cohorts (45,0% and 51,61%). All VC participants were HIV-negative at baseline. Two patients received immunosuppressive therapy, including corticosteroids for sarcoidosis and an anti-TNF antagonist (adalimumab) for psoriasis and ankylosing spondylitis. One patient had urothelial carcinoma.

**Table 2:** Characteristics of the study validation cohort.

Cohort characteristics	Validation cohort			
	TB patients		Healthy controls	
Total number (N)	31		14	
Values	$\bar{\text{x}}$/N	ICR/%	$\bar{\text{x}}$/N	ICR/%
Age (Years)	37.0	26.5 – 43.0	44.5	41.3 – 52.5
Sex (Male)	23	74.19%	2	14.29%
M/XDR therapy	16	51.61%	NA	
Therapy duration (Days)	592	343 -608	NA	
Longer therapy as WHO recommended	12	38.71%	NA	
Smoking	16	51.61%	4	28.57%

### Immune blood cells as features for machine learning scores

**Figure 1:**
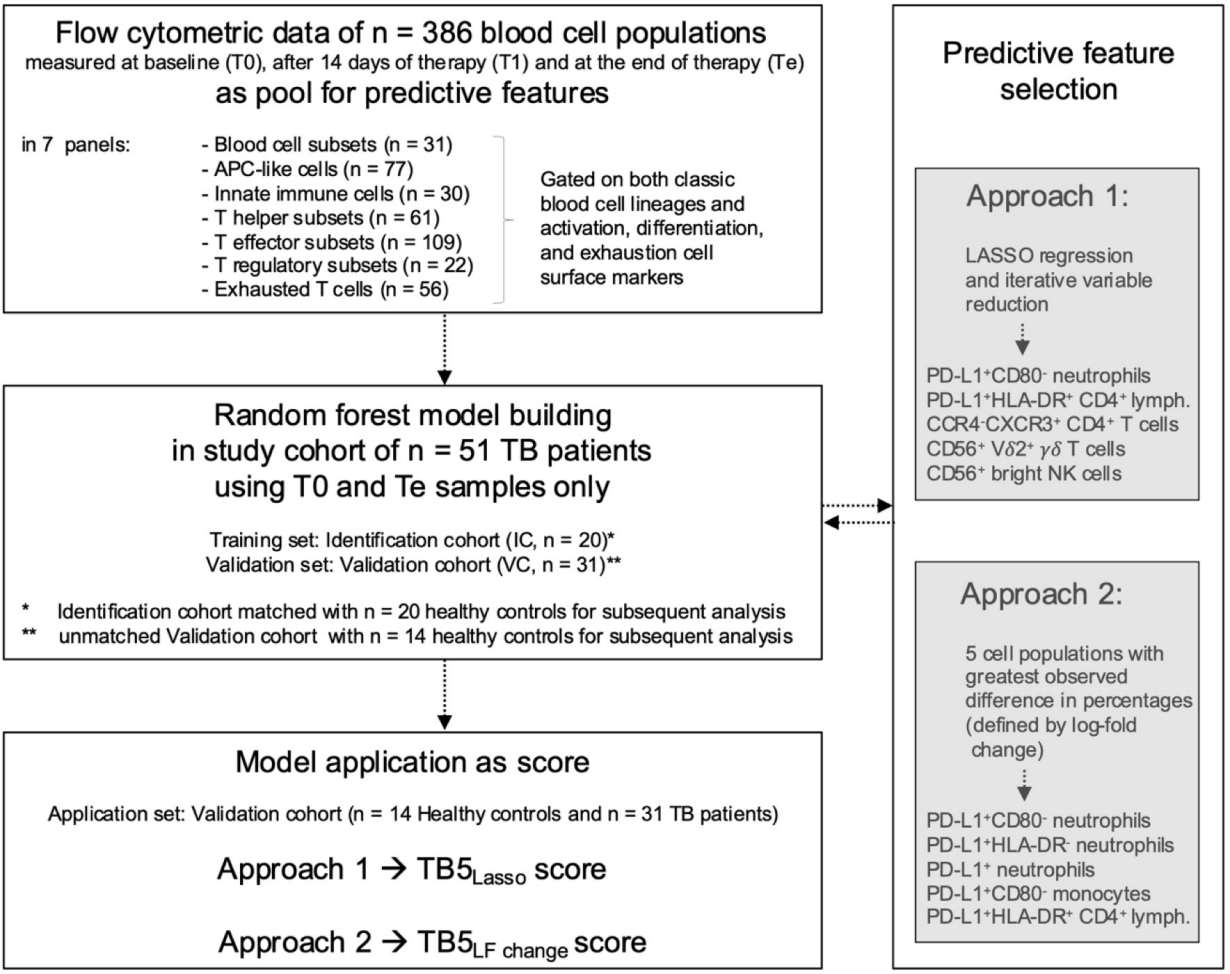
Workflow for developing random forest models and TB5 scores. *Top left*, flow cytometry was performed on 386 blood immune cell populations collected at baseline (T0), after 14 days of therapy (T1), and at the end of therapy (Te) from tuberculosis (TB) patients. Cells were stained using seven distinct panels targeting various immune cell categories, including blood cell subsets (n = 31), APC-like cells (n = 77), innate immune cells (n = 63), T helper subsets (n = 51), T effector subsets (n = 109), T regulatory subsets (n = 36), and exhausted T cells (n = 19). Gating strategies were based on lineage, activation, differentiation, and exhaustion markers. These cell populations were used as input features to develop predictive models. *Middle left*, the random forest model construction used samples from 51 TB patients, which were restricted to the T0 and Te timepoints. The cohort was split into a training set (Identification cohort, IC; n = 20) and a validation set (Validation cohort, VC; n = 31). Healthy controls were only used for subsequent analyzes. *Bottom left*, the probabilities of the models were applied as a score to the validation cohort, including 14 healthy controls and 31 TB patients. *Right*, gray boxes, the two approaches were applied for feature selection. Approach 1 utilized LASSO regression with iterative variable reduction, identifying five predictive cell populations: PD-L1^+^CD80^-^ neutrophils, PD-L1^+^HLA-DR^+^ CD4^+^ lymphocytes, CCR4^-^CXCR3^+^ CD4^+^ T cells, CD56^+^ Vδ2^+^ γδ T cells and CD56^+^ bright NK cells. Approach 2 selected five populations with the largest absolute differences in cell frequencies between T0 and Te, based on log-fold change: PD-L1^+^CD80^-^ neutrophils, PD-L1^+^HLA-DR^+^ neutrophils, PD-L1^+^ neutrophils, PD-L1^+^CD80^-^ monocytes, and PD-L1^+^HLA-DR^+^ CD4^+^ lymphocytes.

A total of 386 blood cell populations were quantified by flow cytometry and served as the input feature set for predictive modeling training of TB status and treatment response in the IC. These cell populations were identified using seven staining panels and represent a comprehensive landscape of innate and adaptive immune responses (**Figure 1**). Classic blood cell lineages (31 cell populations) were gated, along with markers of activation, differentiation, and exhaustion. These subsets included antigen-presenting cells (APCs; n = 77), innate immune cells (n = 30), T helper subsets (n = 61), T effector subsets (n = 109), T regulatory subsets (n = 22) and exhausted T cells (n = 56), with individual gating strategies for the seven staining panels detailed in **Supplementary Figures S1**–**S7**.

Overall, the proportion of missing values was low, with a median missingness of 0% and a mean of 1.35% across variables. The majority of the variables (≥75%) had no missing values, while the maximum missingness observed for any single variable was 21.25%. No clear systematic pattern of missingness was observed. Two approaches were used for feature selection. The first used least absolute shrinkage and selection operator (LASSO) regression with iterative variable reduction. The second approach focused on the cell populations with the largest log-fold changes in percentages between T0 and Te. To allow direct comparison, the log-fold change approach was limited to five cell populations, matching the number selected by LASSO (**Figure 1**). The frequency with which each variable was selected by LASSO was measured. Two of the final model features demonstrated very high selection stability (>95%), indicating robust and reproducible selection across the resampled datasets. The remaining features showed moderate selection frequencies (0.40-0.68), suggesting a contribution to the model with some degree of variability. Overall, this analysis supports the presence of stable underlying signals despite the limited sample size. The model achieved a Brier score of 0.114, indicating good agreement between the predicted probabilities and observed outcomes.

### LASSO-based model identifies five blood cell populations for the best TB treatment discrimination

The random forest model was built using the five features selected by LASSO regression with iterative variable reduction in the training set. The model achieved the highest discrimination accuracy between TB patients before (T0) and after TB treatment (Te) using the following five blood cell populations: PD-L1^+^CD80^-^ neutrophils, PD-L1^+^HLA-DR^+^ CD4^+^ lymphocytes, CCR4^-^CXCR3^+^ CD4^+^ T cells, CD56^+^ Vδ2^+^ γδ T cells and CD56^+^ bright NK cells (**Figure 1**, Approach 1, TB5_Lasso_ score).

### A TB5_LF change_ based model identifies five blood cell populations for the best TB treatment discrimination

We next developed a TB5_LF change_ model to identify the five cell populations with the most pronounced log-fold changes in the percentages of cells between patients at T0 and Te. These included PD-L1^+^CD80^-^ neutrophils and PD-L1^+^HLA-DR^+^ CD4^+^ lymphocytes, which were also identified by LASSO regression. In addition, PD-L1^+^HLA-DR^-^ and PD-L1^+^ neutrophils, as well as PD-L1^+^CD80^-^ monocytes, were identified (**Figure 1**, Approach 2, TB5_LF change_ score).

### Comparative evaluation of the two scores in the validation cohort

**Figure 2:**
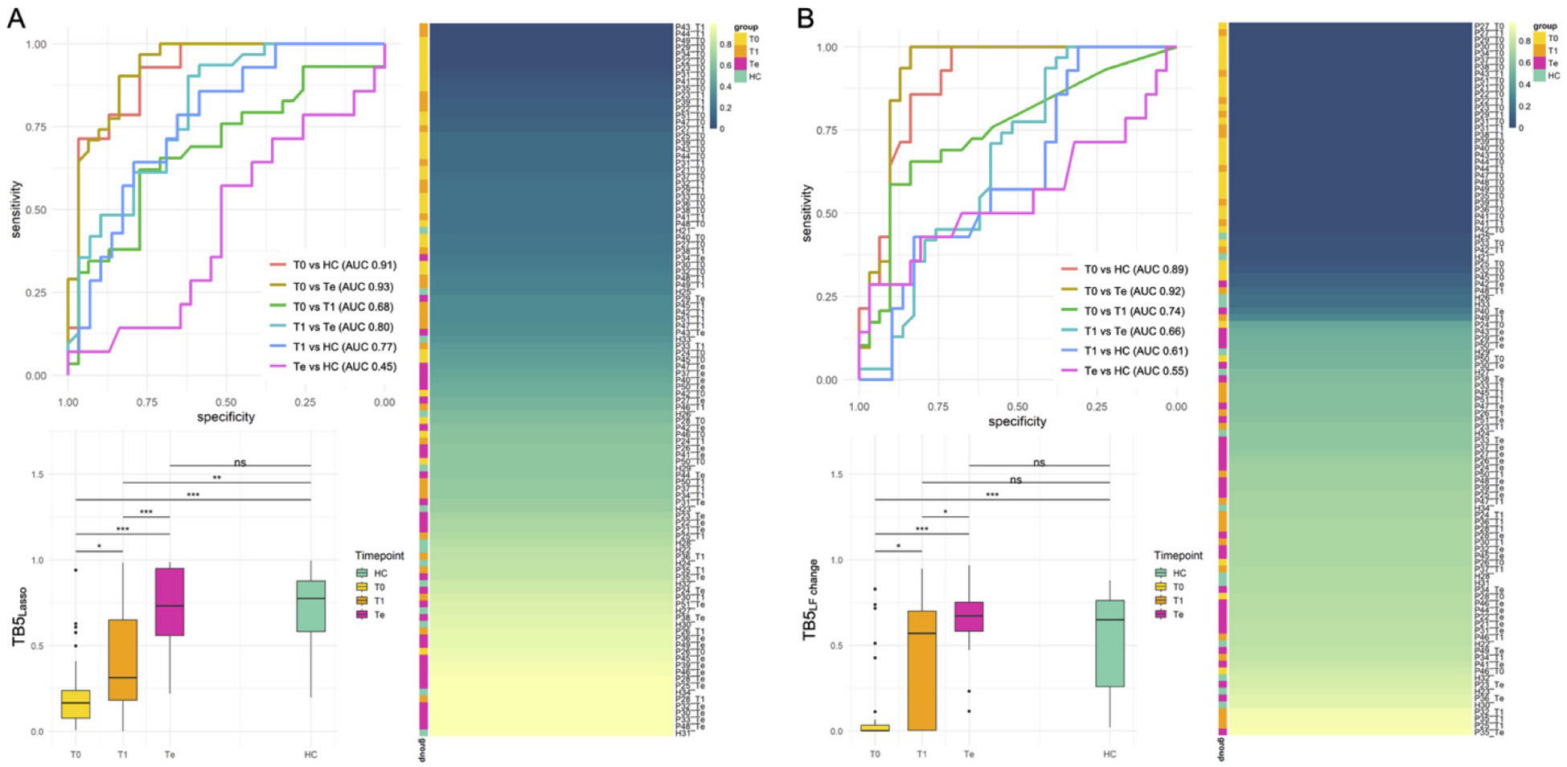
Evaluation of the random forest models. The probabilities of the random forest models were used to calculate scores **(A)** for TB5_Lasso_ score performance in the validation cohort and **(B)** for TB5_LF change_ score performance in the validation cohort. For each **(A, B)**: *Top* left, area under the curve (AUC) values for comparisons between patients at different therapy timepoints, either prior to therapy start (T0), 14 days within therapy (T1), and end of therapy (Te), and healthy controls (HC). *Bottom* left, boxplots of the score at timepoints T0 (yellow), T1 (orange), and Te (pink), as well as in the HC group (green). Statistical significance is indicated as follows: ***0.0001 ≤ p < 0.001; **0.001 ≤ p < 0.01; *0.01 ≤ p < 0.05; ns (not significant) for p ≥ 0.05. *Right*, heatmap of the score. Rows represent individuals at the variable timepoints and healthy controls. Timepoints and HCs are indicated on the left string with T0 (yellow), T1 (orange), Te (pink), and HC (green). Lower scores are primarily observed in therapy-naïve patients (blue end), while higher scores are associated with end-of-therapy timepoints or healthy controls (yellow end).

The probabilities of the random forest models were used to calculate individual scores. The first model, TB5_Lasso_, achieved an out-of-bag (OOB) error rate of 17.5%, indicating good internal model performance. The model discriminated excellently between TB patients before and at the end of therapy (T0 versus Te, AUC 0.93 (0.87–0.99)) and between TB patients and healthy controls (T0 versus HC, AUC 0.91 (0.83–0.99)). The model discrimination was poor during the early treatment phase (T0 versus T1, AUC 0.68 (0.55–0.83)) but had good discrimination between patients early after treatment initiation and at the end of therapy (T1 versus Te, AUC 0.80 (0.69–0.91)) and fair discrimination between T1 and HCs (AUC 0.77 (0.62–0.91)), indicating promising use as a monitoring tool. The model did not discriminate between Te and HCs (AUC 0.45 (0.29–0.64)). The discriminatory ability of the TB5_Lasso_ score progressively increased from T0 to T1, Te, and finally to HCs, as clearly visualized in the heatmap (**Figure 2A**).

The second model, TB5_LF change,_ had a slightly higher OOB error rate of 20%. The discriminative ability between patients before and after TB therapy was also excellent (T0 versus Te, AUC 0.92 (0.83–1)). Likewise, the model had good discrimination between TB patients and healthy controls (T0 versus HC, AUC 0.89 (0.81–0.98)). The model discriminated fairly well between T0 and T1 (AUC 0.74 (0.62–0.87)) and poorly between T1 and both Te and HC (AUC 0.66 (0.51–0.8) and 0.61 (0.44–0.79), respectively). No discriminative ability was observed for Te and HCs (AUC 0.55 (0.34–0.76)). This TB5_LF change_ score showed high variability at early treatment stages but clear separation between T0 and Te (**Figure 2B**). Both TB5 scores at Te were not significantly different from those of the HCs (**Figures 2A, B**).

Overall, the TB5_Lasso_ score demonstrated superior performance in terms of accuracy, early treatment monitoring, and score consistency over time. These findings suggest that multivariate feature selection via LASSO may offer improved robustness for TB patient stratification.

### Machine learning scores correlate with clinical disease severity

**Figure 3:**
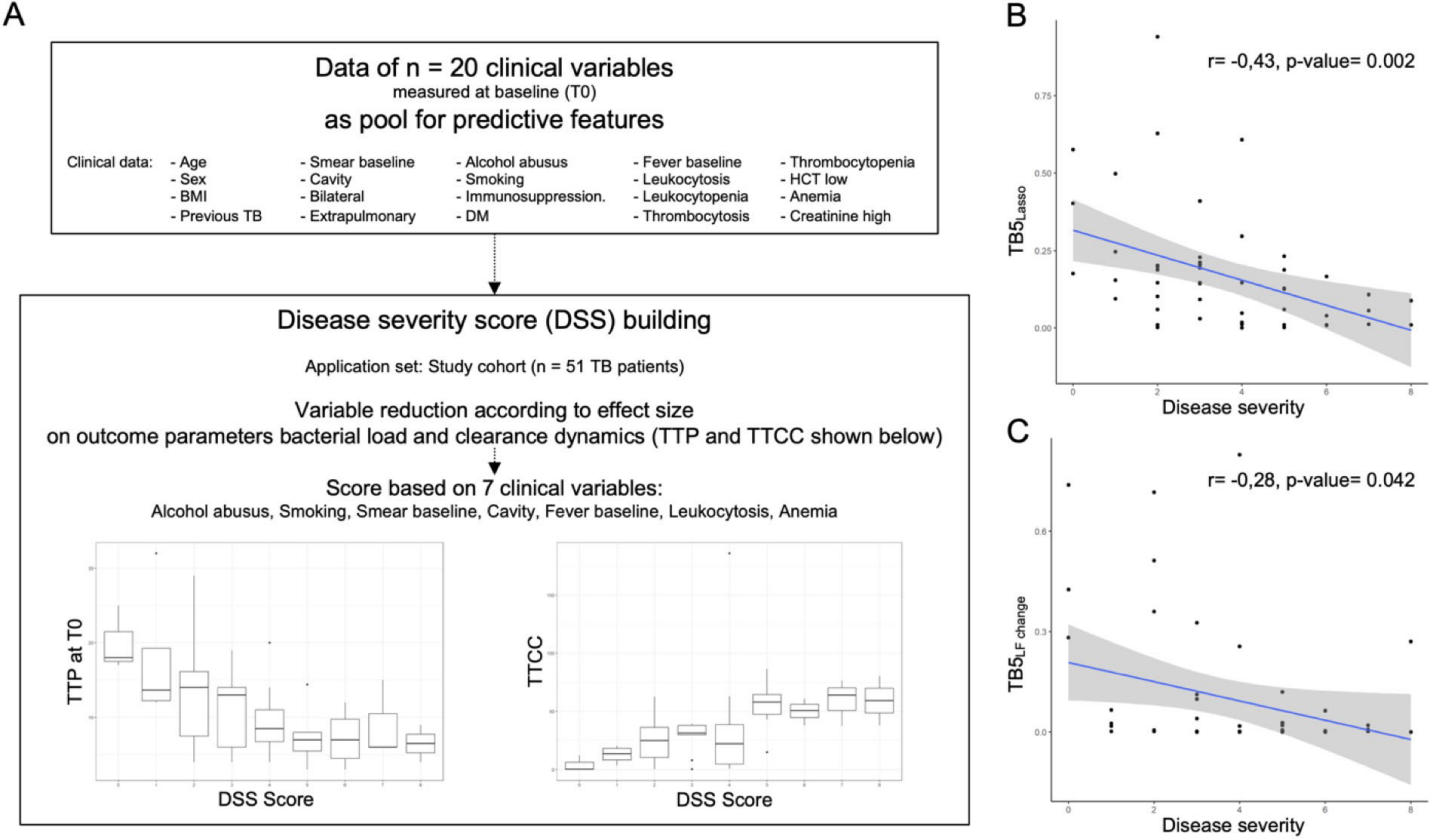
Workflow for development of the descriptive disease severity score (DSS) and its correlation with TB5 scores. **(A)** *Top*, list of clinical variables measured at baseline included in the pool for predictive features used for the development of the DSS. *Bottom*, concise explanation of DSS building based on a simplified point score based on 7 variables. The study participants reached a maximum of 8 out of 9 possible points in the descriptive disease severity score. Boxplots showing the association between DSS and clinical endpoints time to positivity at baseline (TTP at T0) and time to culture conversion (TTCC): Higher DSS values are associated with a shorter TTP at baseline and a longer TTCC. **(B)** Correlations between the TB5_Lasso_ score and DSS in the complete study cohort (n = 51 TB patients) at baseline. **(C)** Correlations between the TB5_LF change_ score and DSS in the complete study cohort (n = 51 TB patients) at baseline. In both cases, a lower TB5 score is associated with increased disease severity.

We next evaluated the association between the TB5 scores and disease severity across the complete study cohorts, including both the identification and validation cohorts. Among the 20 baseline clinical variables, seven were associated with culture-related disease severity: alcohol abuse, active smoking, high smear grade, lung cavities, fever, leukocytosis, and anemia (**Figure 3A**). On the basis of these variables, we constructed an exploratory simplified composite disease severity score (DSS) derived from a descriptive model that could subsequently stratify TB patients according to bacterial load and clearance dynamics (**Figure 3A**). The TB5_Lasso_ score correlated moderately with the composite disease severity score (r=-0.43; p=0.002), while the TB5_LF change_ score showed a weaker but significant correlation (r=-0.28; p=0.042; **Figures 3B, C**).

### Added value of machine learning over classical analysis

**Figure 4:**
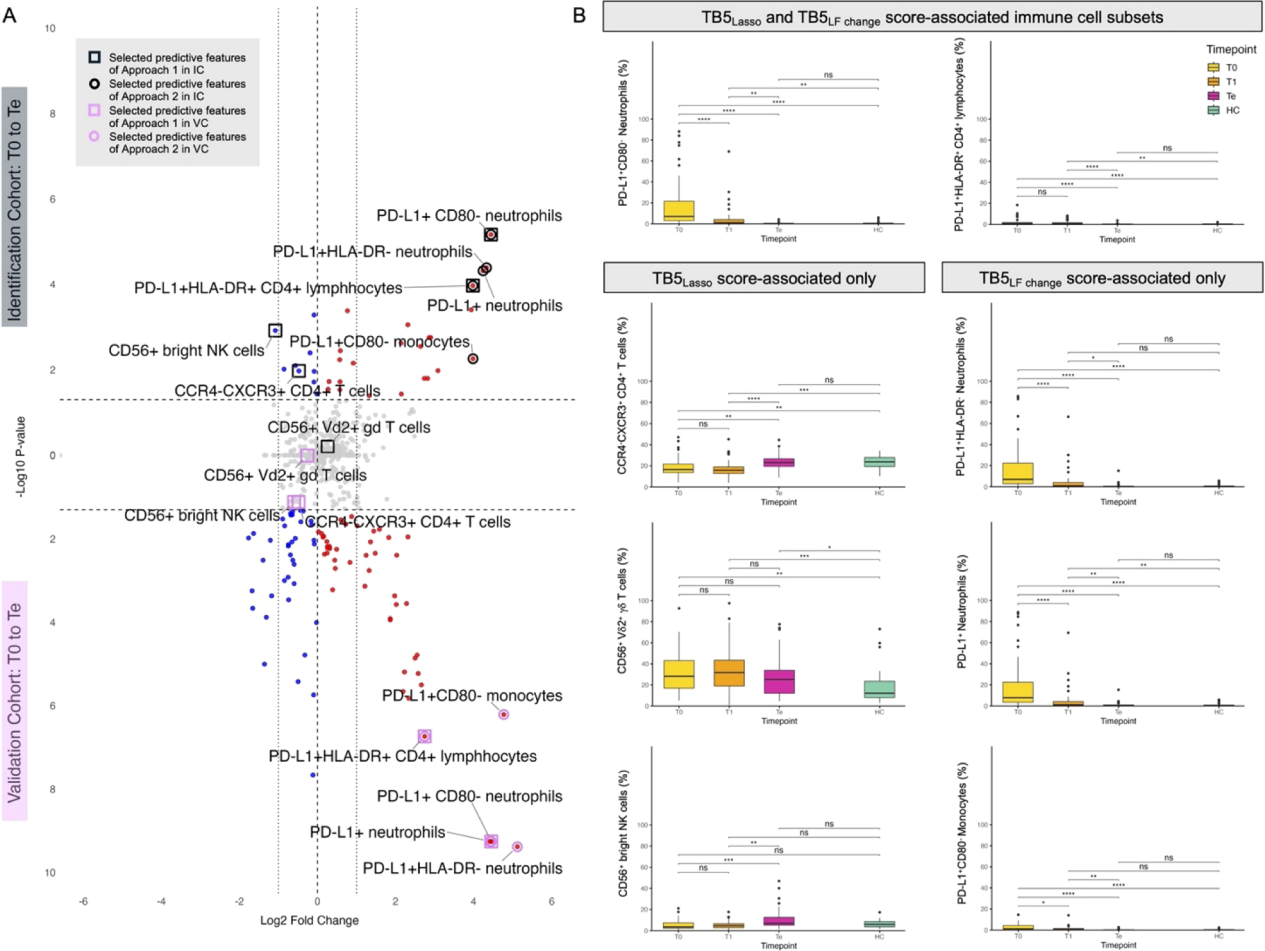
Differential analysis of the TB5 score-associated immune cell subsets. **(A)** Combined volcano plot of the differential percentages of all 386 immune cell populations measured prior to therapy start (T0) and at the end of therapy (Te). The identification cohort is shown at the top, and the validation cohort is shown at the bottom. Statistical comparisons among T0 and Te were calculated using the Benjamini-Hochberg-(BH)corrected Wilcoxon rank-sum test. The x-axis indicates the fold change. Squares indicate populations included in the TB5_Lasso_ score and circles denote cell populations with the highest fold change, which contribute to the TB5_LF change_ score. γδ T cells are represented as gd T cells in the figure. **(B)** Boxplots of TB5 score-associated immune cell subset frequencies across longitudinal and control timepoints including the complete study cohort. *Top*, populations common to both TB5_Lasso_ and TB5_LF change_. *Bottom left*, populations unique to the TB5_Lasso_ score. *Bottom right*, populations unique to the TB5_LF change_ score. The frequencies of the eight immune cell populations were compared across three longitudinal timepoints, namely, baseline (T0, yellow), 14 days of therapy (T1, orange) and therapy end (Te, pink) and a healthy control (HC, green) group. Each boxplot displays the distribution of cell frequency (%) per group, with boxes indicating the interquartile range (IQR), horizontal lines representing medians, and whiskers extending to 1.5xIQR. The y-axis indicates the percentage of cells and is capped at 100% to reflect the biological scale; significance bars may extend beyond 100% to maintain spacing. The x-axis shows the timepoints. Statistical comparisons among the T0, T1, Te and HC groups were calculated using Bonferroni-corrected Wilcoxon rank-sum tests. P values are indicated as follows: ****p < 0.0001; ***0.0001 ≤ p < 0.001; **0.001 ≤ p < 0.01; *0.01 ≤ p < 0.05; ns (not significant) for p ≥ 0.05.

To assess the added value of our machine learning approach, we examined how the cell populations selected by the two modeling strategies (Lasso and log fold-change) appeared when analyzed using conventional differential methods. Specifically, we visualized the distribution and significance of these individual populations using mirrored volcano plots of the IC and VC (**Figure 4A**). To determine their value in univariate comparisons, we analyzed each of the selected cell populations in HC and TB patients from the complete study cohort, including the dynamic changes in each population over time until the end of treatment (**Figure 4B**).

PD-L1^+^CD80^-^ neutrophils and PD-L1^+^HLA-DR^+^ CD4^+^ lymphocytes were the two cell populations selected by both modeling strategies and showed significant changes between T0 and Te with conventional evaluation, with normalization at Te compared with HCs (**Figure 4B**).

In addition to the shared populations, the three remaining populations selected by the log fold-change approach, PD-L1^+^HLA-DR^-^ neutrophils, PD-L1^+^CD80^-^ monocytes, and PD-L1^+^ neutrophils, showed some of the most pronounced differences between T0 and Te in classical differential analysis (**Supplementary Table 2**). These populations, along with other PD-L1-expressing antigen-presenting cells and lymphocytes, were markedly elevated at baseline and declined substantially with treatment, highlighting their association with active disease in the early phase. The consistent behavior of the cells across patients at T0 and Te suggests that PD-L1 may reflect underlying immune regulatory processes in TB. While these populations were not selected by the LASSO model - likely owing to redundancy or high correlation - they may still be of interest as candidate biomarkers. Notably, at T0 and T1, we observed considerable interindividual variability in PD-L1+ APCs and lymphocyte percentages, suggesting differences in immune status and that only a subset of TB patients exhibit early normalization. This limits the ability to monitor treatment response during the first 14 days of therapy in our study cohort.

The three additional cell populations selected exclusively by LASSO regression, namely, CCR4^-^CXCR3^+^ CD4^+^ T cells, CD56^+^ bright NK cells, and CD56^+^ Vδ2^+^ γδ T cells, did not rank among the top features according to conventional univariate analysis. This highlights the advantage of multivariate modeling in uncovering immune signatures with high predictive value that may not show the largest fold changes or significance. CCR4^-^CXCR3^+^ CD4^+^ T cells and CD56^+^ bright NK cells displayed statistically significant changes between T0 and Te despite the modest fold changes in expression and normalized to the levels observed in HCs. In contrast, the percentage of CD56^+^ Vδ2^+^ γδ T cells changed little during therapy but remained significantly different from that in HCs at the Te stage.

Together, these findings underscore the strengths of machine learning in detecting complex, treatment-relevant immune dynamics that classical approaches may overlook.

## Discussion

Our study introduces an exploratory immune profiling approach and identifies novel immune signatures relevant to TB. We developed and evaluated two predictive scores via machine learning and mathematical modeling of longitudinal flow cytometric data of 386 cell populations from peripheral whole blood: The TB5_Lasso_ and the TB5_LF change_ score. The TB5_Lasso_ score exhibited superior discriminatory performance, effectively differentiating TB patients at baseline and at the end of therapy, as well as from healthy individuals. These findings suggest that the TB5_Lasso_ score may capture an immune signature of potential relevance for future diagnostic and monitoring applications, including patient stratification (e.g., by disease severity). In contrast, the TB5_LF change_ score showed limited performance for early-stage therapy monitoring, especially because of high interindividual variability in the marker PD-L1.

Regardless of the selection method, two of the five cell populations were identical in both the TB5_Lasso_ and the TB5_LF change_ score, namely, PD-L1^+^CD80^-^ neutrophils and PD-L1^+^HLA-DR^+^ CD4^+^ lymphocytes. The PD1/PD-L1 pathway is an immune checkpoint that plays a critical regulatory role in the immune response in TB through the inhibition of T-cell effector functions (11, 12). Pathogens use this pathway to evade immune detection, resulting in persistent infections (13). While this mechanism may protect infected tissues from immune-mediated damage, it compromises the effectiveness of the immune response and limits the ability of the host to clear the infection (13, 14). Neutrophils are the most abundant phagocytic cells in the airways of patients with active pulmonary TB and serve as critical first responders in the immune system (15). Increased PD-L1 expression on neutrophils is associated with active TB, and successful antimycobacterial therapy has been shown to reduce PD-L1 levels to those observed in healthy individuals (16). In a mouse study, transient expression of CD80 was detected on neutrophils that had internalized *M. tuberculosis*, suggesting a functional link between mycobacterial uptake and costimulatory marker expression. CD80^-^ neutrophils are the predominant subset of lung neutrophils during TB infection and are less effective at inducing dendritic cell maturation or T-cell activation than their more immunologically active CD80^+^ counterparts are (17). Beyond neutrophils, adaptive immune cells such as CD4^+^ lymphocytes, especially CD4^+^ T cells, are essential for protective immunity against TB, as they coordinate immune responses and cytokine signaling (18, 19). In the absence of direct staining of the T-cell receptor (TCR) complex, we cannot exclude the possibility that the CD4^+^ lymphocyte population may also include unconventional CD3^-^ CD4^+^ cells. While PD-L1^+^ expression is commonly associated with antigen-presenting cells, both PD-L1 and its receptor PD1 are also expressed on CD4^+^ T cells and are linked to impaired T-cell function (20). Unlike that on APCs, PD-L1 expression remains persistently elevated on subsets of CD4^+^ T cells, potentially contributing to ongoing immune modulation even during recovery (21). HLA-DR expression is a well-established marker in active TB, indicating recent T-cell activation and proliferation, and is associated with effector phenotypes (22). The presence of PD-L1^+^ HLA-DR^+^ CD4^+^ lymphocytes during active disease suggests immune dysregulation, as these cells simultaneously exhibit signs of activation and exhaustion.

In addition to these shared populations, the TB5_Lasso_ score included CCR4^-^CXCR3^+^ CD4^+^ T cells, CD56^+^ Vδ2^+^ γδ T cells and CD56^+^ bright NK cells. CCR4 and CXCR3 are chemokine receptors involved in directing T-cell trafficking. CCR4^-^CXCR3^+^ CD4^+^ T cells exhibit a Th1 phenotype, characterized by proinflammatory cytokine production and orchestration of the cellular immune response to *M. tuberculosis* (23). Vδ2^+^ γδ T cells play important roles in infection prior to the development of a robust conventional T-cell response, bridging innate and adaptive immunity (24, 25). The presence of CD56^+^ Vδ2^+^ γδ T cells may reflect early cytotoxic activity. Similarly, CD56^+^ bright NK cells are recognized for their potent cytokine production ability (26, 27). Overall, these findings suggest that cell populations associated with PD-L1-mediated immune exhaustion (neutrophils and CD4^+^ lymphocytes), early cytotoxic mycobacterial control (CD56^+^ Vδ2^+^ γδ T cells and CD56^+^ bright NK cells), and a Th1 immune response (CCR4^-^CXCR3^+^) constitute a comprehensive immunological signature that varies with disease and treatment stage. These additional markers in the TB5_Lasso_ score, particularly those reflecting cytotoxic and Th1 responses, may explain its enhanced sensitivity in both early and late-stage disease differentiation. This integrated signature identifies candidate biomarkers and provides a basis for hypothesis generation regarding their potential role in TB diagnosis and treatment monitoring, warranting validation in larger and more diverse cohorts in future research.

PD-L1^+^ neutrophils (PD-L1^+^CD80^-^, PD-L1^+^HLA-DR^-^ and PD-L1^+^), monocytes (PD-L1^+^CD80^-^), and CD4^+^ lymphocytes (PD-L1^+^HLA-DR^+^) were key populations identified through predictive feature selection for the TB5_LF change_ score. The percentage of cells carrying the ligand PD-L1 markedly differed between patients at the beginning and end of therapy, highlighting its potential as a marker for both the diagnosis of TB and monitoring treatment response. However, the high variability in the percentages of PD-L1^+^ cells after 14 days of therapy limits their performance at early treatment stages. This interindividual variability warrants further investigation in future studies. HLA-DR^-^ neutrophils are also abundant in TB, represent immature subsets likely derived from emergency granulopoiesis, and are associated with active disease status and functional impairment (28). Both the expression of costimulatory CD80 and HLA-DR on neutrophils has been linked to the acquisition of antigen-presenting functions (29). The expression of PD-L1 on neutrophils lacking CD80 or HLA-DR suggests a regulatory, suppressive role that potentially dampens immune activation and facilitates immune evasion by *M. tuberculosis*, similar to the characteristics of myeloid-derived suppressor cells (MDSCs) (30). Furthermore, PD-L1 levels on CD14^+^ monocytes are known to be significantly elevated in individuals with active TB compared with those with latent TB infection or noninfected individuals. Notably, increased PD-L1 expression is correlated with increased bacterial burden (31). This expression decreases significantly after two months of anti-TB therapy, again suggesting its potential as a biomarker for both disease severity and treatment monitoring (31).

Our study has several limitations. A technical limitation is the complexity of the high-dimensional, multiparameter flow cytometry panel used in this exploratory study, which limits its clinical applicability. A simplified panel comprising a limited set of key cell populations identified here will be necessary to facilitate translation into clinical practice. As a sampling limitation, the study cohort was relatively small in scale, lacked inclusion of patients with unrelated pulmonary diseases, and was conducted at a single clinical site, which may limit the generalizability of the findings. The machine learning models were trained on a specific dataset, and their predictive accuracy may differ across external validation cohorts. Given the relatively small size of the identification cohort in relation to the number of initially assessed variables, there is a potential risk of overfitting. To mitigate this, we applied LASSO regression with cross-validation for feature selection and further reduced the model using AIC and variable importance to obtain a parsimonious set of predictors. In addition, the model was evaluated in an independent validation cohort, which supports its generalizability. Nevertheless, overfitting might still occur, and the findings should be interpreted with caution and require confirmation in larger, independent datasets. Model calibration in the validation cohort indicated good agreement between the predicted probabilities and observed outcomes. However, given the limited sample size of the validation cohort, these results should be interpreted with caution. While we accounted for confounding factors such as age, sex, and smoking status, residual confounding could still have affected the results. The sex and age distribution of the validation cohort differed from those of the training cohort, which could introduce confounding effects since TB responses may vary by demographic factors. Moreover, the fact that the scores remained informative under these imbalances suggests a degree of robustness. Nevertheless, confirmation in larger, more balanced cohorts is needed to separate disease effects from demographic influences. The study excluded people living with HIV or those with immunosuppression, which limits the applicability of the findings to these populations. While we observed correlations between bacterial load, clearance dynamics, and composite measures of disease severity, whether a higher bacterial burden or prolonged time to culture conversion directly translate into clinically worse outcomes for patients remains unknown. Our analysis relies on surrogate markers of severity rather than definitive clinical endpoints such as treatment failure, relapse, or mortality. Consequently, the associations we report should be interpreted as suggestive rather than causal. Future prospective studies with long-term clinical follow-up are needed to establish whether bacterial load and culture conversion kinetics are true predictors of adverse patient outcomes.

Our findings support the growing interest in whole blood-based biomarker research in TB, advancing beyond traditional single-marker approaches by integrating high-dimensional flow cytometry with machine learning. This strategy enhances diagnostic specificity and sensitivity through advanced feature selection and predictive modeling. Future research should validate the potential diagnostic and monitoring accuracy of the newly identified cell populations in the TB5 scores using simplified flow cytometry panels with a limited set of key populations. Assessing the feasibility of point-of-care implementation is essential for maximizing clinical application and impact. Our data also provide insights into TB-related immunopathology, which may help identify targets to reduce post-TB morbidity and mortality. The incorporation of multiomic data and the performance of longitudinal studies could further refine model accuracy and provide more in-depth insight into TB pathophysiology.

## Data Availability

The raw data supporting the conclusions of this article will be made available by the authors, without undue reservation.

## Group members of the DZIF-TB cohort study group

Korkut Avsar, Georg P. Glattki, Torsten Goldmann, Gunar Günther, Michael Hoelscher, Elmira Ibraim, Stefan HE. Kaufmann, Irina Kontsevaya, Florian P. Maurer, Marius Müller, Dörte Nitschkowski, Ioana D. Olaru, Christina Popa, Andrea Rachow, Thierry Rolling, Jan Rybniker, Helmut JF. Salzer, Maren Schuhmann, Victor Spinu, Isabelle Suárez, Elena Terhalle, Markus Unnewehr, Frank van Leth, January Weiner 3rd.
