# Supplementary material for: PD-L1-centric whole blood-based immune signature profiles of tuberculosis patients during therapy
Source: Front Immunol. 2026 Jun 12;17:1829438. doi: 10.3389/fimmu.2026.1829438 (PMC13305727; doi:10.3389/fimmu.2026.1829438)
Supplement: Supplementary file 1 [file SupplementaryFile1.docx]

**Supplementary information**

**Supplementary Tables and Figures**

Title: PD-L1-centric whole blood-based immune signature profiles of tuberculosis patients during therapy

Johanna Eggeling^1,2,3,4,5*^, Martina Sester^6^, Christoph Lange^1,2,3,7^, Jan Heyckendorf^8,9^, Barbara Kalsdorf^1,2,3^, Anna M. Mandalakas^1,2,7^, Andrew R. DiNardo^7^, David Lewinsohn^10^, Dagmar Schaub^1,2,3^, Tina Schmidt^6^, Eva Tolosa^11^, Maja Reimann^1,2,3^†, Patricia M. Sánchez Carballo^1,2,3*^† for the DZIF-TB cohort study group‡

^1^Research Center Borstel, Leibniz Lung Center, Division of Clinical Infectious Diseases; Borstel, Germany

^2^German Center for Infection Research (DZIF), Tuberculosis Unit, Partner Site Hamburg-Lübeck-Borstel-Riems; Germany

^3^Lübeck University, Respiratory Medicine & International Health; Lübeck, Germany

^4^Schleswig-Holstein University Hospital, Pediatric Oncology and Rheumatology; Kiel, Germany

^5^Schleswig-Holstein University Hospital, Neonatology, Pediatric Pneumology and Neuropediatrics; Kiel, Germany,

^6^Saarland University, Department of Transplant and Infection Immunology; Homburg, Germany

^7^Baylor College of Medicine and Texas Children´s Hospital, Global Tuberculosis Programm, Division of Global Health; Houston, Texas, USA

^8^Schleswig-Holstein University Hospital, Internal Medicine I, Leibniz Lung Clinic; Kiel, Germany

^9^Airway Research Center North (ARCN), German Center for Lung Research (DZL); Germany

^10^Oregon Health and Science University, Pulmonary, Allergy and Critical Care Medicine, Portland; Oregon, USA

^11^ University Medical Center Hamburg-Eppendorf, Institute of Immunology, Hamburg Center for Translational Immunology; Hamburg, Germany

‡Additional members of the DZIF-TB cohort study group: Korkut Avsar, Georg P. Glattki, Torsten Goldmann, Gunar Günther, Michael Hoelscher, Elmira Ibraim, Stefan HE. Kaufmann, Irina Kontsevaya, Florian P. Maurer, Marius Müller, Dörte Nitschkowski, Ioana D. Olaru, Christina Popa, Andrea Rachow, Thierry Rolling, Jan Rybniker, Helmut JF. Salzer, Maren Schuhmann, Victor Spinu, Isabelle Suárez, Elena Terhalle, Markus Unnewehr, Frank van Leth, January Weiner 3rd.

# Supplementary Tables

## Supplementary Table S1

**Table S1:** **Antibody-fluorochrome combinations and reagent details.** Summary of antibodies used across the seven staining panels for multicolor flow cytometric analysis. For each marker, the table lists the fluorochrome conjugate, antibody clone, dilution factor, and supplier with catalog number. The listed combinations were optimized for simultaneous surface staining to ensure minimal spectral overlap. All antibodies were titrated prior to use to determine optimal staining concentrations.

| Panel | Fluorochrome | Clone | Dilution factor | Supplier and Catalog number |
| --- | --- | --- | --- | --- |
| 1:  Blood cell subsets | anti-CD14-BV421 | M5E2 | 1:3 | BioLegend, US, 301830 |
|  | anti-CD4-HorizonV500 | PRA-T4 | 1:3 | BD, US, 560768 |
|  | anti-CD8-BV605 | RPA-T8 | 1:30 | BioLegend, US, 301040 |
|  | anti-CD19-FITC | HIB19 | 1:30 | BioLegend, US, 302206 |
|  | anti-TCRγδ-PE | 11F2 | 1:3 | BD, US, 333141 |
|  | anti-CD3-PerCPCy5.5 | UCHT1 | 1:30 | BioLegend, US, 300430 |
|  | anti-CD56-PECy7 | MEM-188 | 1:3 | BioLegend, US, 304628 |
|  | anti-CD45-APC | HI30 | 1:30 | BioLegend, US, 304037 |
|  | anti-CD16-APCCy7 | 3G8 | 1:100 | BioLegend, US, 302018 |
| 2:  APC-like cells | anti-HLA-DR-PacificBlue | L243 | 1:30 | BioLegend, US, 307633 |
|  | anti-CD4-HorizonV500 | PRA-T4 | 1:3 | BD, US, 560768 |
|  | anti-CD8-BV605 | RPA-T8 | 1:30 | BioLegend, US, 301040 |
|  | anti-CD19-FITC | HIB19 | 1:30 | BioLegend, US, 302206 |
|  | anti-CD274-PE | 29E2A3 | 1:30 | BioLegend, US, 329706 |
|  | anti-CD14-PerCPCy5.5 | M5E2 | 1:3 | BioLegend, US, 301824 |
|  | anti-CD80-PECy7 | L307.4 | 1:3 | BD, US, 561135 |
|  | anti-CD45-APC | HI30 | 1:30 | BioLegend, US, 304037 |
|  | anti-CD16-APCCy7 | 3G8 | 1:100 | BioLegend, US, 302018 |
| 3:  Innate immune cells | anti-CD161-BV421 | HP-3G10 | 1:10 | BioLegend, US, 339914 |
|  | anti-CD4-HorizonV500 | PRA-T4 | 1:3 | BD, US, 560768 |
|  | anti-CD8-BV605 | RPA-T8 | 1:30 | BioLegend, US, 301040 |
|  | anti-TCRVδ2-FITC | IMMU 389 | 1:10 | Beckman Coulter, US, IM1464 |
|  | anti-TCRγδ-PE | 11F2 | 1:3 | BD, US, 333141 |
|  | anti-CD3-PerCPCy5.5 | UCHT1 | 1:30 | BioLegend, US, 300430 |
|  | anti-CD56-PECy7 | MEM-188 | 1:3 | BioLegend, US, 304628 |
|  | anti-TCRVα7.2-APC | 3C10 | 1:100 | BioLegend, US, 351708 |
|  | anti-CD69-APCCy7 | FN50 | 1:3 | BioLegend, US, 310914 |
| 4:  T helper subsets | anti-CD161-BV421 | HP-3G10 | 1:10 | BioLegend, US, 339914, |
|  | anti-CD4-HorizonV500 | PRA-T4 | 1:3 | BD, US, 560768 |
|  | anti-CD8-BV605 | RPA-T8 | 1:30 | BioLegend, US, 301040 |
|  | anti-CD183-AF488 | G025H7 | 1:10 | BioLegend, US, 353710 |
|  | anti-CD196-PE | G034E3 | 1:100 | BioLegend, US, 353410 |
|  | anti-CD3-PerCPCy5.5 | UCHT1 | 1:30 | BioLegend, US, 300430 |
|  | anti-CD194-PECy7 | L291H4 | 1:10 | BioLegend, US, 359410 |
|  | anti-CD197-AF647 | 150503 | 1:3 | BD, US, 560816 |
|  | anti-CD69-APCCy7 | FN50 | 1:3 | BioLegend, US, 310914 |
| 5:  T effector subsets | anti-CD57-PacificBlue | HNK-1 | 1:30 | BioLegend, US, 359608 |
|  | anti-CD4-HorizonV500 | PRA-T4 | 1:3 | BD, US, 560768 |
|  | anti-CD8-BV605 | RPA-T8 | 1:30 | BioLegend, US, 301040 |
|  | anti-CD45RO-FITC | UCHL1 | 1:2.5 | BioLegend, US, 304204 |
|  | anti-TCRγδ-PE | 11F2 | 1:3 | BD, US, 333141 |
|  | anti-CD3-PerCPCy5.5 | UCHT1 | 1:30 | BioLegend, US, 300430 |
|  | anti-CD28-PECy7 | CD28.2 | 1:100 | BioLegend, US, 302926 |
|  | anti-CD197-AF647 | 150503 | 1:3 | BD, US, 560816 |
|  | anti-CD27-APCCy7 | O323 | 1:3 | BioLegend, US, 302816 |
| 6:  T regulat. subsets | anti-HLA-DR-PacificBlue | L243 | 1:30 | BioLegend, US, 307633 |
|  | anti-CD4-HorizonV500 | PRA-T4 | 1:3 | BD, US, 560768 |
|  | anti-CD8-BV605 | RPA-T8 | 1:30 | BioLegend, US, 301040 |
|  | anti-CD45RO-FITC | UCHL1 | 1:2.5 | BioLegend, US, 304204 |
|  | anti-CD25-PE | BC96 | 1:3 | BioLegend, US, 302606 |
|  | anti-CD3-PerCPCy5.5 | UCHT1 | 1:30 | BioLegend, US, 300430 |
|  | anti-CD39-PECy7 | A1 | 1:100 | BioLegend, US, 328212 |
|  | anti-CD127-AF647 | HIL-7R-M21 | 1:10 | BD, US, 558598 |
|  | anti-CD279-APCCy7 | EH122H7 | 1:10 | BioLegend, US, 329922 |
| 7:  Exhausted T cells | anti-HLA-DR-PacificBlue | L243 | 1:30 | BioLegend, US, 307633 |
|  | anti-CD4-HorizonV500 | PRA-T4 | 1:3 | BD, US, 560768 |
|  | anti-CD8-BV605 | RPA-T8 | 1:30 | BioLegend, US, 301040 |
|  | anti-CD45RO-FITC | UCHL1 | 1:2.5 | BioLegend, US, 304204 |
|  | anti-Tim-3-PE | 344823 | 1:3 | R&D Systems, US, FAB2365P |
|  | anti-CD3-PerCPCy5.5 | UCHT1 | 1:30 | BioLegend, US, 300430 |
|  | anti-CD28-PECy7 | CD28.2 | 1:100 | BioLegend, US, 302926 |
|  | anti-CD197-AF647 | 150503 | 1:3 | BD, US, 560816 |
|  | anti-CD279-APCCy7 | EH122H7 | 1:10 | BioLegend, US, 329922 |

## Supplementary Table S2

**Table S2:** **Immune cell subset frequencies and statistical comparisons across conditions.** Summary of the relative frequencies of selected immune cell subsets measured at three time points/conditions (T0, T1, Te) and in healthy controls (HC). Medians (m, %), Bonferroni-adjusted p values from pairwise Wilcoxon rank sum tests (T0, T1, Te, and HC), and corresponding log fold changes for the indicated comparisons are shown. Data are grouped according to associations with TB5 scores.

# ****Supplementary Figures****

## Supplementary Figure S1


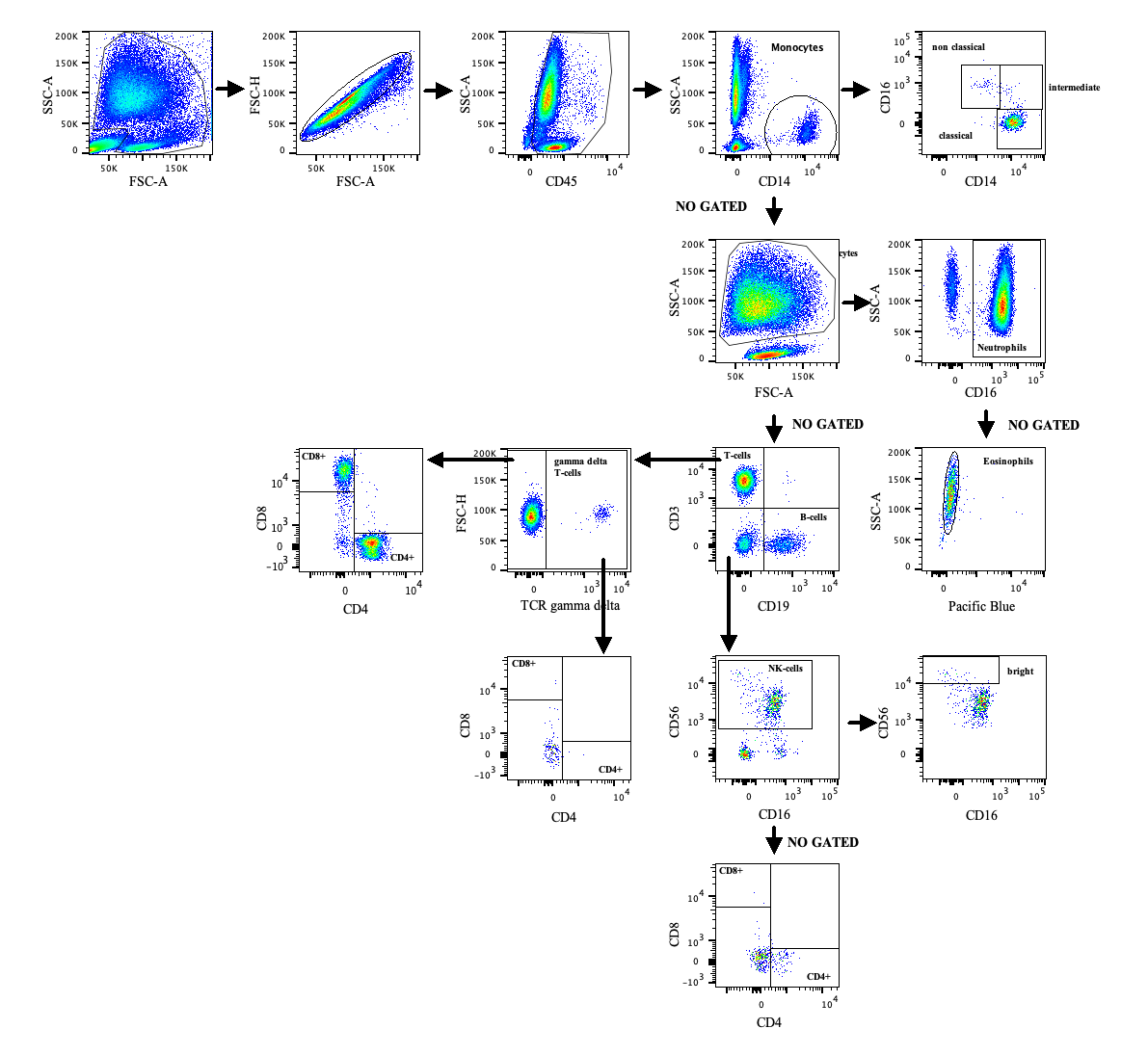


**Figure S1: Gating strategy for identification of classic blood cell lineages (panel 1).** Cell debris was excluded based on forward and side scatter area (FSC-A vs SSC-A), and doublets were excluded using forward scatter height versus area (FSC-H vs FSC-A). Leukocytes were identified based on CD45 expression (CD45+). Monocytes were defined as CD14+ (CD14 vs SSC-A) and further classified into classical (CD14+CD16−), intermediate (CD14+CD16+), and non-classical (CD14dimCD16+) subsets. CD45+ non-monocyte populations were divided into granulocytes and lymphocytes. Granulocytes were subdivided by CD16 expression into CD16+ (neutrophils) and CD16− subsets. Lymphocytes were further gated into T cells (CD19−CD3+), B cells (CD19+CD3−), and NK cells (CD19−CD3−CD56+). T cells were categorized by TCRγδ expression into γδ T cells and non-γδ T cells. Non-γδ T cells were further subdivided based on CD4 and CD8 expression into CD4−CD8+, CD4+CD8−, and CD4−CD8− (double negative, DN) subsets. NK cells were stratified into CD56+ (NKdim) and CD56++ (NKbright) populations.

## Supplementary Figure S2


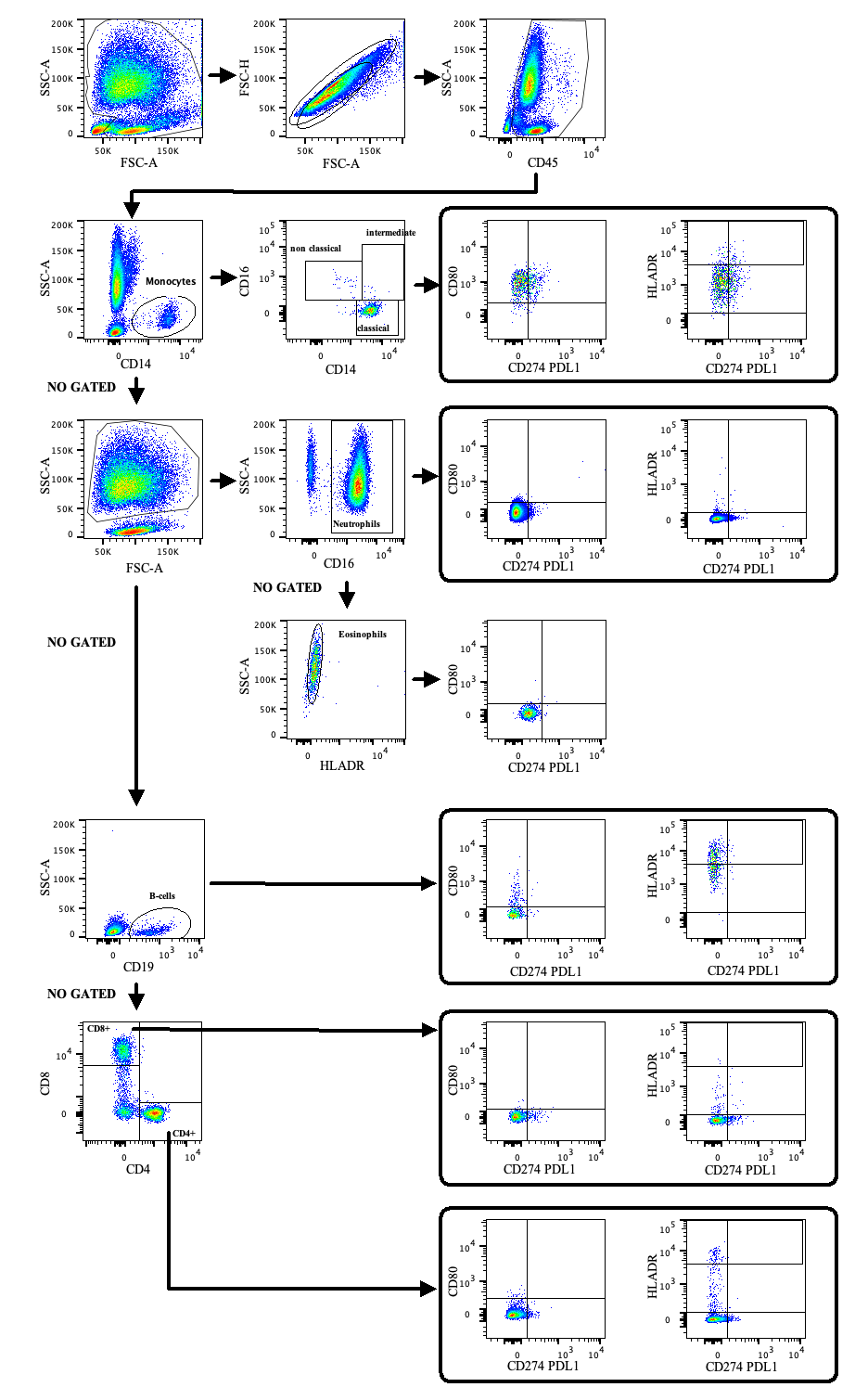


**Figure S2: Gating strategy for identification of antigen-presenting cells (panel 2).** Cell debris was excluded based on forward and side scatter area (FSC-A vs SSC-A), and doublets were excluded using forward scatter height versus area (FSC-H vs FSC-A). Leukocytes were identified based on CD45 expression (CD45+). Monocytes were identified by area in the sideward scatter (SSC-A) and by CD14 expression and further classified into classical (CD14+CD16−), intermediate (CD14+CD16+), and non-classical (CD14dimCD16+) subsets. CD45+ non-monocyte populations were divided into granulocytes and lymphocytes. Granulocytes were gated by high area in the sideward scatter (SSC-A) and separated into neutrophils (CD16+) and eosinophils (HLA-DR-). B cells were identified as CD19+ lymphocytes. T cells were identified among the CD19− population and further subdivided based on CD4 and CD8 expression into CD4+ T cells, CD8+ T cells, and CD4−CD8− double-negative (DN) T cells. Each immune cell subset was subsequently analyzed for the expression of CD80 and PD-L1 (CD274), as well as HLA-DR.

## Supplementary Figure S3


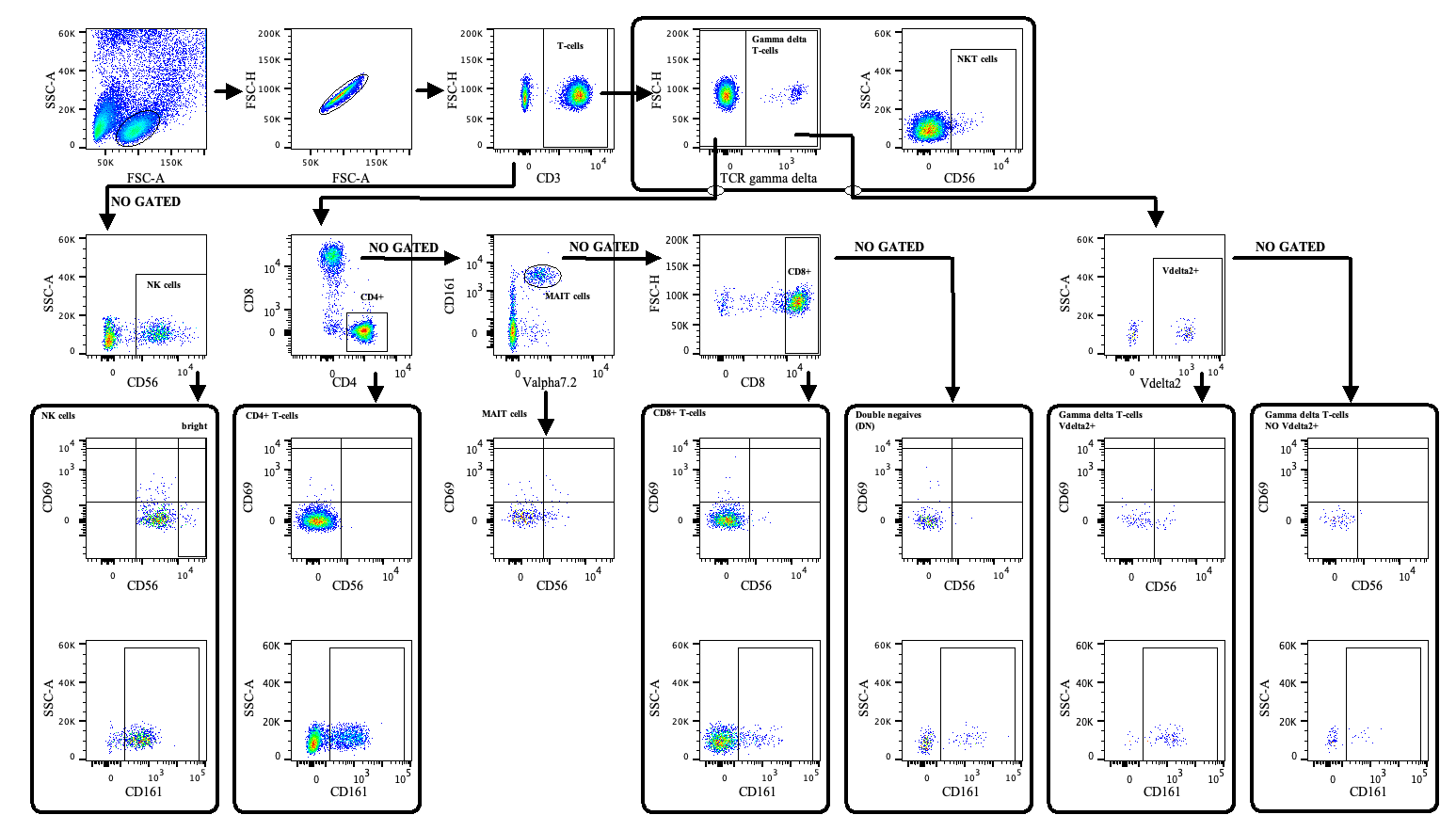


**Figure S3: Gating strategy for identification of innate immune cells (panel 3).** Lymphocytes were identified by area in the forward and sideward scatter plot (FSC-A vs. SSC-A). Doublets were excluded using forward scatter area versus highth (FSC-A vs FSC-H). CD3+ T cells were selected and categorized by TCRγδ expression into γδ T cells and non-γδ T cells. γδ T cells were further subdivided into Vδ2+ and Vδ2− subsets. Additional CD3+ T cell subsets were defined with a downstream analysis in the non-γδ T cells: First, CD4+ T cells were identified. MAIT cells were defined as non-CD4 T cells by Vα7.2 and CD161 expression (Vα7.2+ CD161+). Further, non-MAIT cells were divided into CD8+ T cells (CD8+CD4−) and double-negative (DN) T cells (CD4−CD8−). NK cells were gated from the CD3− population as CD56+ lymphocytes and stratified into CD56bright and CD56dim subsets. NKT-like cells were defined as CD3+CD56+ cells. All subsets were assessed for surface expression of CD69, CD56, and CD161.

## Supplementary Figure S4


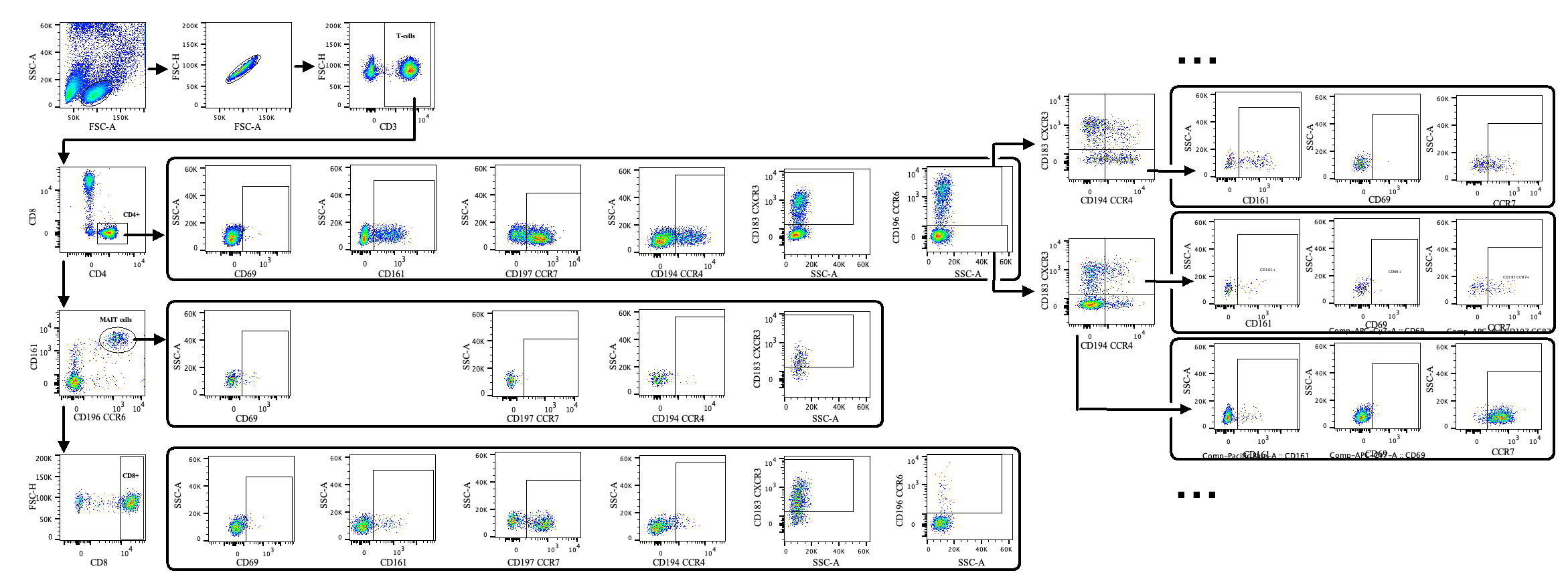


**Figure S4: Gating strategy for identification of T helper subsets (panel 4).** Lymphocytes were identified by area in the forward and sideward scatter plot (FSC-A vs. SSC-A). Doublets were excluded using forward scatter area versus highth (FSC-A vs FSC-H). CD3^+^ T cells were selected and categorized by CD4 and CD8 expression. CD4+CD8- T cells were further analyzed for the surface markers CCR6, CCR4 and CXCR3 to define helper T cell subsets, e.g. Th1 (CXCR3⁺CCR4⁻CCR6⁻), Th1/Th17 (CXCR3⁺CCR4⁻CCR6⁺), and Th17 (CXCR3⁻CCR4⁺CCR6⁺CD161⁺). The CD3⁺CD4⁻ population was analyzed for MAIT cells, defined as CCR6⁺CD161high. Remaining non-MAIT CD3⁺CD4⁻ cells are gated into CD8^+^ T cells. Cell subsets were assessed for surface expression of for CD69, CD161, CCR7, CCR4, CXCR3, and CCR6.

## Supplementary Figure S5


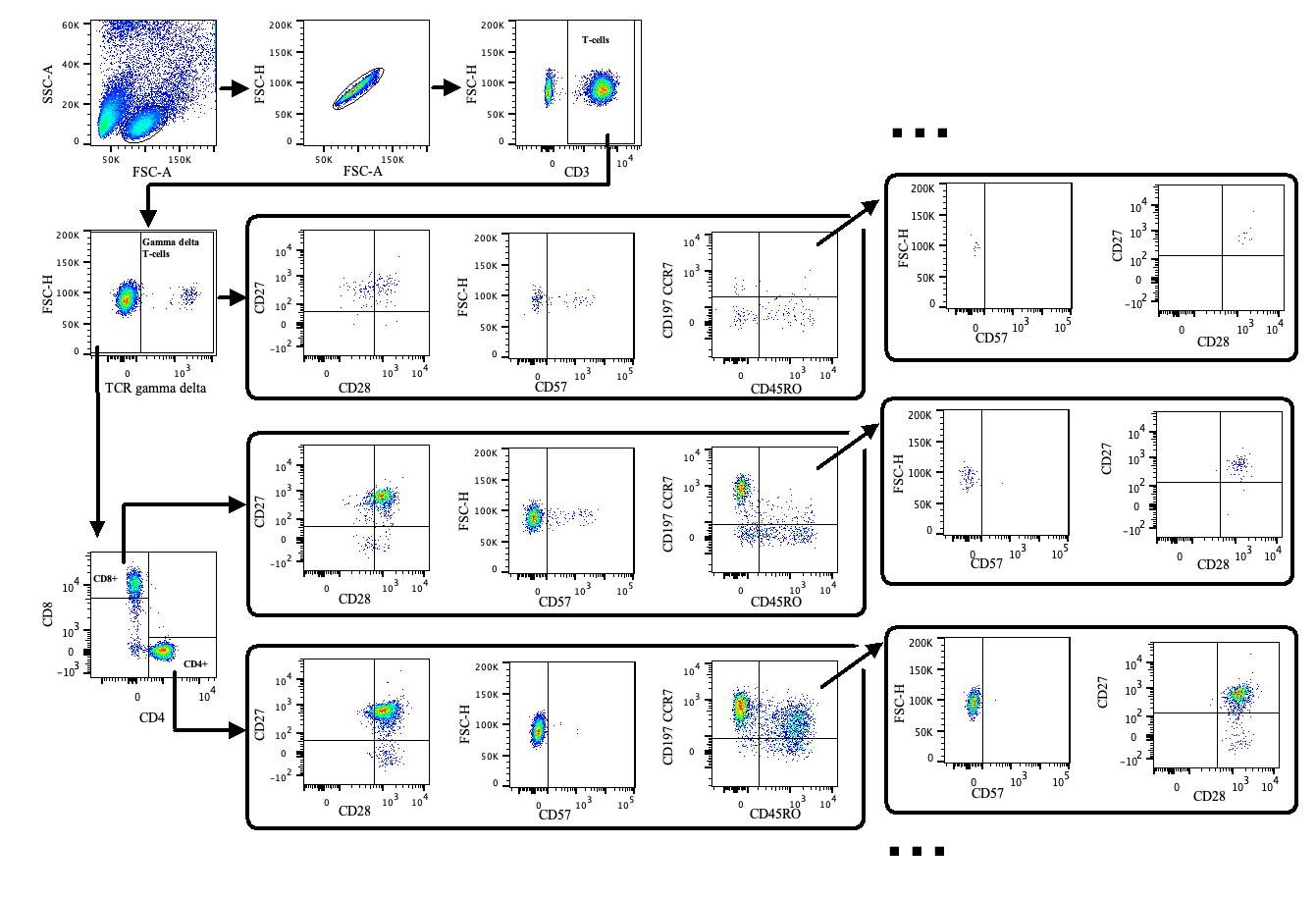


**Figure S5: Gating strategy for identification of T effector subsets (panel 5).** Lymphocytes were identified by area in the forward and sideward scatter plot (FSC-A vs. SSC-A). Doublets were excluded using forward scatter area versus highth (FSC-A vs FSC-H). CD3^+^ T cells were selected and categorized by TCRγδ expression into γδ T cells and non-γδ T cells. Non-γδ T cells were further subdivided into CD4^+^CD8^-^ and CD4^-^CD8^+^ T cells. Both γδ T cells and T helper and cytotoxic subsets were again subdivided into memory phenotypes based on CD45RO and CCR7 expression: Effector memory (EM: CD45RO⁺CCR7⁻), terminal effector memory (TEM: CD45RO⁻CCR7⁻), central memory (CM: CD45RO⁺CCR7⁺), and naïve T cells (N: CD45RO⁻CCR7⁺). Cell subsets were analyzed for the expression of CD28, CD27, and CD57.

## Supplementary Figure S6


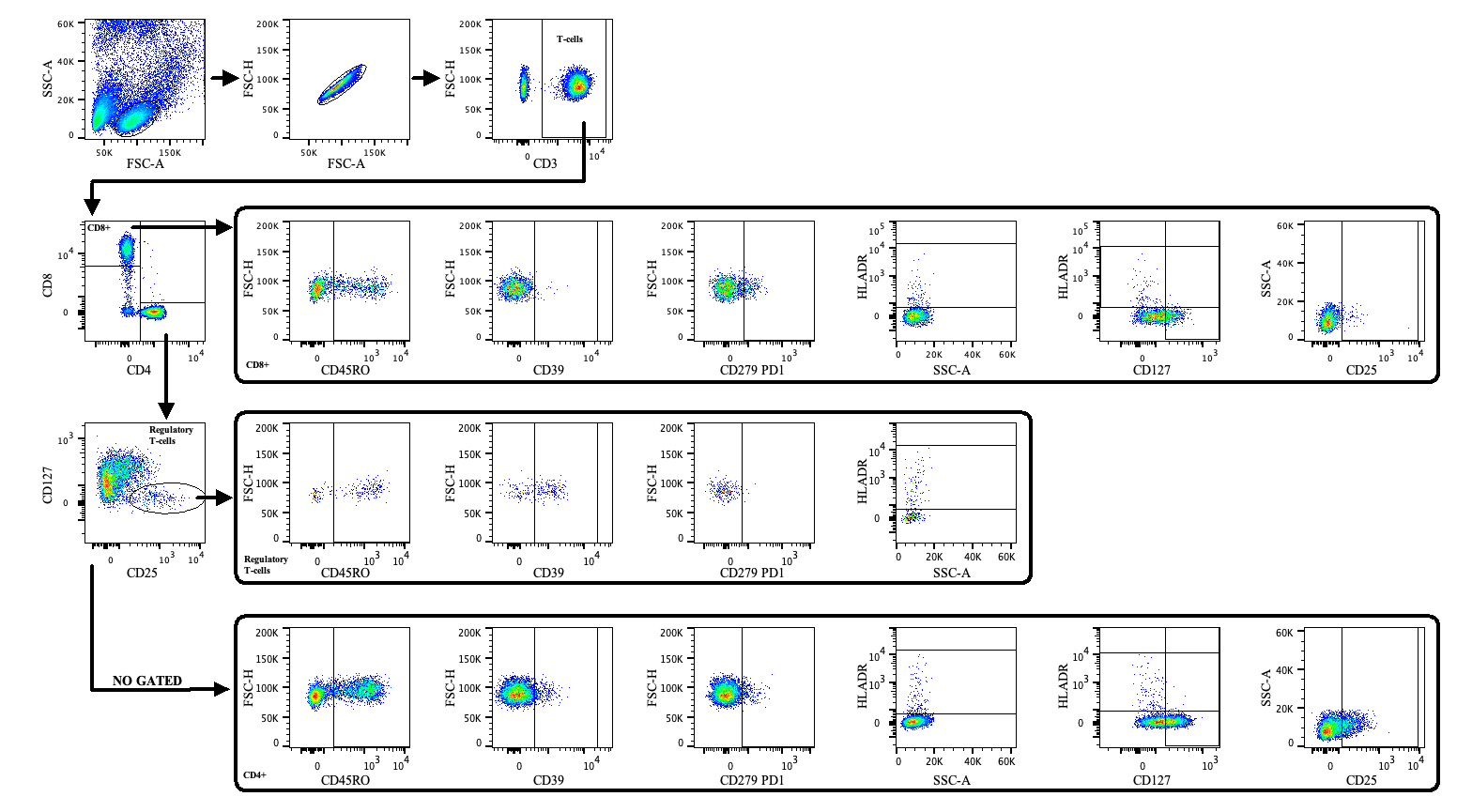


**Figure S6: Gating strategy for identification of T regulatory subsets (panel 6).** Lymphocytes were identified by area in the forward and sideward scatter plot (FSC-A vs. SSC-A). Doublets were excluded using forward scatter area versus highth (FSC-A vs FSC-H). CD3^+^ T cells were selected and categorized by CD4 and CD8 expression into CD4^+^CD8^-^ and CD4^-^CD8^+^ T cells. Within CD4⁺CD8⁻ T cells, T regulatory cells were defined as CD127⁻CD25high. Cell subsets were analyzed for the expression of CD45RO, CD39, PD-1 (CD279), HLA-DR, CD127, and CD25.

## Supplementary Figure S7


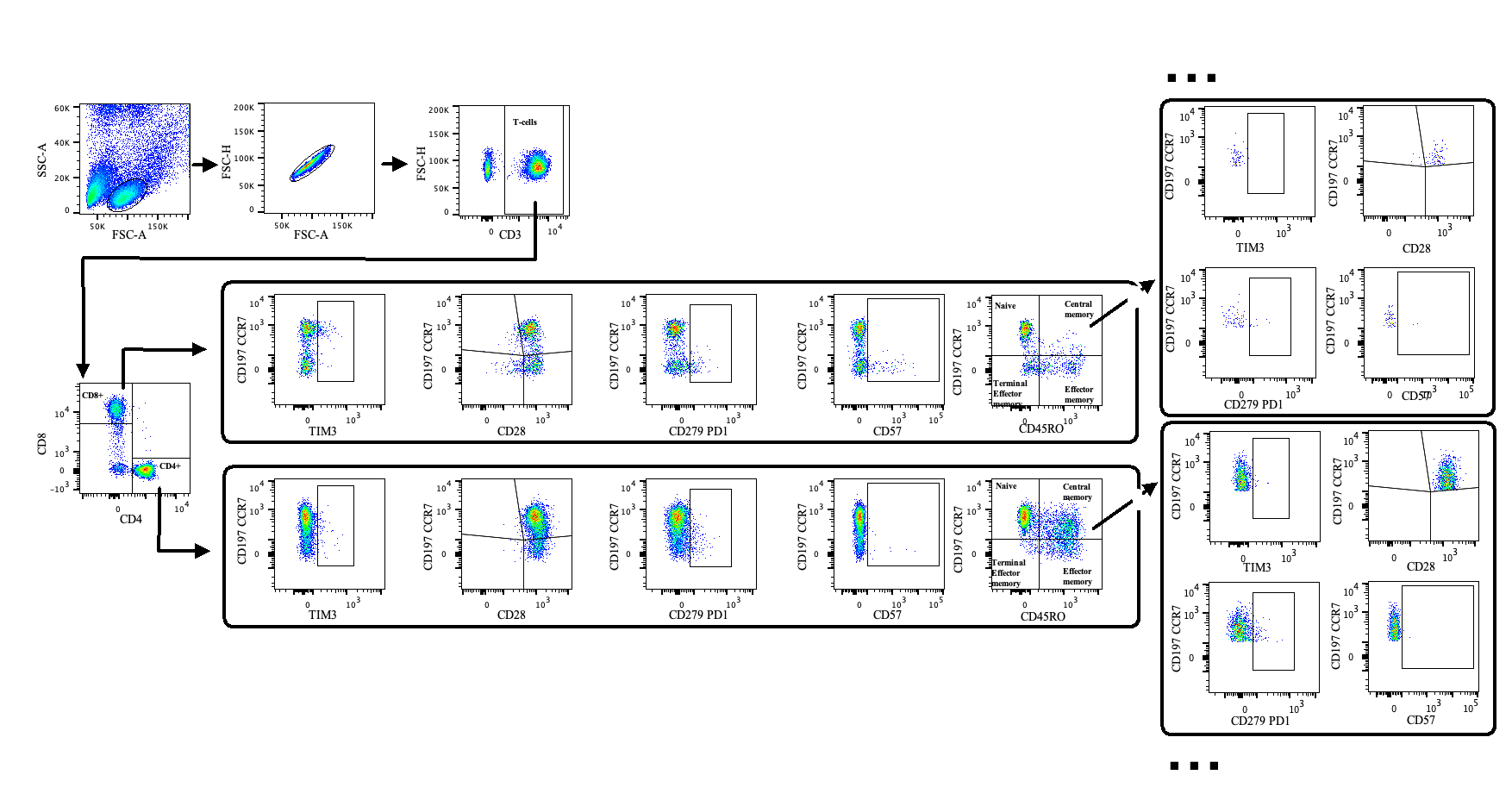


**Figure S7: Gating strategy for identification of exhausted T cells (panel 7).** Lymphocytes were identified by area in the forward and sideward scatter plot (FSC-A vs. SSC-A). Doublets were excluded using forward scatter area versus highth (FSC-A vs FSC-H). CD3^+^ T cells were selected and categorized by CD4 and CD8 expression into CD4^+^CD8^-^ and CD4^-^CD8^+^ T cells. These were again subdivided into memory phenotypes based on CD45RO and CCR7 expression: Effector memory (EM: CD45RO⁺CCR7⁻), terminal effector memory (TEM: CD45RO⁻CCR7⁻), central memory (CM: CD45RO⁺CCR7⁺), and naïve T cells (N: CD45RO⁻CCR7⁺). Cell subsets were analyzed for the expression of TIM3, CD28, PD-1 (CD279), and CD57.

## Supplementary Figure S8

**Figure S8: Workflow for model development and validation.** Schematic overview of the analytical pipeline used for model construction and evaluation. The identification (training) cohort was first defined, followed by missing data imputation and feature processing. Feature selection was performed using least absolute shrinkage and selection operator (LASSO) regression, and a random forest model was subsequently applied. Variables were further reduced to derive a final 5-variable model. This model was then applied to an independent validation cohort, and its performance was assessed using area under the receiver operating characteristic curve (AUC) analysis.
